# Hemoglobin Q-Iran detected in family members from Northern Iran: a case report

**DOI:** 10.1186/1752-1947-6-47

**Published:** 2012-02-06

**Authors:** Mohammad Khorshidi, Payam Roshan, Nooshin Bayat, Mohammad Reza Mahdavi, Hossein Najmabadi

**Affiliations:** 1 Thalassemia Research Center, Mazandaran University of Medical Sciences, Sari, Iran; 2 Research Department, Fajr Laboratory Center, Keshavarz Boulevard, Sari, Iran; 3 Kariminejad-Najmabadi Pathology & Genetics Center, 2 Medical Building, 4th Street, Hassan Seyf Street, Shahrak Gharb, Tehran, PO Box: 14665/154, Iran

## Abstract

**Introduction:**

Hemoglobin Q-Iran (α75Asp→His) is an important member of the hemoglobin Q family, molecularly characterized by the replacement of aspartic acid by histidine. The first report of hemoglobin Q-Iran and the nomenclature of this hemoglobinopathy dates back to 1970. Iran is known as a country with a high prevalence of α- and β-thalassemia and different types of hemoglobinopathy. Many of these variants are yet to be identified as the practice of molecular laboratory techniques is limited in this part of the world. Applying such molecular methods, we report the first hemoglobin Q-Iran cases in Northern Iran.

**Case presentation:**

An unusual band was detected in an isoelectric focusing test and cellulose acetate electrophoresis of a sample from a 22-year-old Iranian man from Mazandaran Province. Capillary zone electrophoresis analysis identified this band as hemoglobin Q. A similar band was also detected in his mother's electrophoresis (38 years, Iranian ethnicity). The cases underwent molecular investigation and the presence of a hemoglobin Q-Iran mutation was confirmed by the amplification refractory mutation system polymerase chain reaction method. Direct conventional sequencing revealed a single guanine to cytosine missense mutation (c.226G > C; *G*AC >*C*AC) at codon 75 in the α-globin gene in both cases.

**Conclusion:**

The wide spectrum and high frequency of nondeletional α-globin mutations in Mazandaran Province is remarkable and seem to differ considerably from what has been found in Mediterranean populations. This short communication reports the first cases of patients with hemoglobin Q found in that region.

## Introduction

Hemoglobin (Hb) Q is a single nucleotide polymorphism occurring in the Hb α-2 chain. Hb Q variants are recognized by a slow-moving band migrating at a similar position to Hb S on alkaline pH electrophoresis. This Hb Q variant has normal solubility [1].

A number of important members of the Hb Q family share a certain molecular feature-the replacement of aspartic acid (Asp) by histidine (His) at different positions in the amino acid chain. These include Hb Q-Thailand (α74 Asp→His), Q-India (α64 Asp→His) and Q-Iran (α75 Asp→His). The association of Hb Q-Thailand with α-thalassemia is frequently reported and the majority of patients show moderate red cell microcytosis [2]. In contrast, there are few reports of any affiliation of Hb Q-India and Hb Q-Iran with other Hb disorders and deletions [3].

Computerized simulation of secondary and tertiary protein structures of these Hb molecules by standard bioinformatic methods suggest that Hb Q-Iran and Hb Q-Thailand have one and two extra helices respectively, whilst Hb Q-India has a protein structure similar to the normal Hb molecule [4]. In the heterozygous state, patients with Hb Q-India or Hb Q-Iran do not have the thalassemia phenotype or any distinctive clinical manifestation. Furthermore, these Hb abnormalities do not affect hematologic features. The replacement of aspartic acid with histidine is on the surface of the protein structure and does not affect the protein interchain contacts and electrical charges of the molecule, and therefore does not cause any changes in hematologic parameters and indices [5]. Interestingly, in a unique report of a homozygous patient with Hb Q-Iran, no clinical symptom was observed [6].

The first report of Hb Q-Iran and the nomenclature of this hemoglobinopathy dates back to 1970 and the work of Lorkin *et al*., introducing the substitution of aspartic acid by histidine at position α75 (EF4) as the responsible defect for this hemoglobinopathy [5]. In 2007, Rahimi and colleagues reported the first incidence of such a hemoglobinopathy in an Iranian individual [7].

Iran is known as a country with a high prevalence of α- and β-thalassemia and different types of hemoglobinopathy. Many of these variants are yet to be identified as the practice of molecular laboratory techniques is limited in this part of the world. Applying molecular methods, we here report the first cases of patients with Hb Q-Iran in Northern Iran.

## Case presentation

A 22-year-old Iranian man originally from the northern province of Mazandaran, was admitted to Fajr Laboratory Center (Sari, Iran) for premarital thalassemia screening. All evaluated hematologic indices were in the normal range (Table 1); however, both isoelectric focusing and cellulose acetate electrophoresis techniques revealed an unusual slow moving electrophoretic band in the location of Hb Q. A hematological investigation was then performed for the whole family. His father's blood tableau and Hb electrophoretic pattern were completely normal. His mother was a 38-year-old of Iranian ethnicity with normal hematologic indices. Her Hb electrophoretic pattern contained a band suspected to be Hb Q. Further evaluation by automatic capillary zone electrophoresis (Minicap system, Sebia, France) revealed an extra peak in the graphs of our two patients, identified as Hb Q (Table 1). Previous reports of the quantity of Hb Q-Iran are stated to be 17% to 19% of the total Hb content. In our study, we found the amount of Hb Q-Iran to be 19.2% in these heterozygous individuals.

**Table 1:** Hematologic indices and Hb electrophoresis of our patients.

	Proband	Mother
Sex	Male	Female
Age (years)	22	38
Red blood cells (× 10^6^/μL)	5.69	4.56
Hb (g/dL)	15.7	12.6
Hematocrit (%)	46.9	37.2
Mean corpuscular volume	82.4	81.7
MCH	27.7	27.6
MCH concentration (%)	33.6	33.8
Hb A (%)	78.2	78.3
Hb A2 (%)	2.1	2
Hb A2 variant (%)	0.5	0.5
Hb Q (%)	19.2	19.2

Hb: hemoglobin; MCH: Mean corpuscular hemoglobin

**Figure 1:**
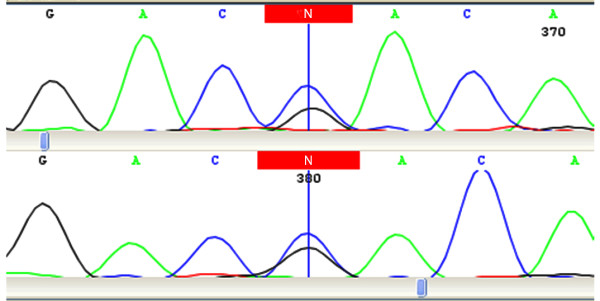
**Deoxyribonucleic acid sequence analysis of the α2-globin gene**. The coding exon deoxyribonucleic acid from our patient was amplified by polymerase chain reaction and sequenced. A segment of the nucleotide sequences containing the mutation is shown. The position of the single guanine to cytosine missense mutation (codon 75 G > C) is indicated by the colored box.

The family was then referred to the Kariminejad-Najmabadi Pathology and Genetics Center for molecular analysis. Deoxyribonucleic acid (DNA) samples were obtained from all members of the family and analyzed for α-globin mutations. The samples were examined by DNA analysis for the presence of the Hb Q-Iran mutation using the amplification refractory mutation system polymerase chain reaction method. Automated direct nucleotide sequencing (ABII377, Applied Biosystems, Foster City, California, USA) of the amplified α2- and α1-globin genes was performed to characterize other nondeletional α-thalassemia determinants. Direct conventional sequencing revealed a single guanine to cytosine missense mutation (c.226G > C; *G*AC >*C*AC) at codon 75 in the α-globin gene in all samples except from the father (Figure 1). No other α-globin mutation could be detected by direct nucleotide sequencing.

Despite the normal clinical features of our patients, laboratory findings led to the identification of a rare Hb variant, Hb Q-Iran. Hereby, we report a family in the north of Iran whose members are carriers of the Hb Q-Iran gene.

## Discussion

Iran is a multiethnic country with a vast variation of thalassemia-causing mutations and there are reports of hemoglobinopathies from different regions of that country [8]. The northern (Mazandaran and Gilan) and southern (Khuzestan) provinces of Iran are the areas most prone to different Hb abnormalities and there have been several studies for identification of these variants in these regions [9]. Mazandaran is a province in the north of Iran, by the Caspian Sea. The wide spectrum and high frequency of nondeletional α-globin mutations in Mazandaran Province is remarkable and seems to differ considerably from what has been found in Mediterranean populations [9].

We have previously reported of less common Hb α-chain mutations in that region, namely initiation codon mutation (ATG > AGG, α2), J-Paris-I (GCC > GAC, α1), codon 14 mutation (TGG > TAG, α1), codon 22 mutation (GGC > GGT, α2), Caserta (GCG > ACG, α2), intervening sequence (IVS)-I-116 (A > G, α2), IVS-II-55 (T > G, α2), Bleuland (ACC > AAC, α2), Sun Prairie (GCT > CCT, α2), 3'UTR nt 46 (C > A, α2) [9], J-Toronto (GCC > GAC, α1) and Setif (GAC > TAC, α2) [10]. This short communication reports the first Hb Q cases found in that region.

Besides Iran, there have been several reports of Hb-Q from Turkey [3,6]. This is not the first hemoglobinopathy in common between Iranian and Turkish populations. There are several findings of different abnormal Hb molecules such as Hb J-Iran, Hb D-Iran, Hb Hamadan and Hb D-Punjab in people of both ethnicities [11]. These findings, along with further haplotype studies, may suggest a common ancestral origin for the people in that region and help to propose immigration patterns at the time of colonization by Aryans on the Iranian Plateau and in Middle Asia, circa 4,000 BC.

## Conclusion

Different hemoglobinopathies are frequently reported from Northern Iran, but this is the first report of an Hb Q-Iran mutation in members of a family from that region. This report provides a substantial piece of evidence in completing the list of discovered Hb abnormalities on the southern shores of the Caspian Sea.

## Consent

Written informed consent was obtained from the patient and his mother for publication of this case report and any accompanying images. A copy of the written consent is available for review by the Editor-in-Chief of this journal.

## Competing interests

The authors declare that they have no competing interests.

## Authors' contributions

MK performed the clinical examination of the patients and introduced the cases. PR performed the laboratory work and analysis and wrote the case reports. NB carried out the molecular laboratory work. MRM carried out the laboratory work and analysis of the data. HN performed the molecular laboratory work, wrote the molecular section and revised the case reports. Hereby all authors verify that they read and approved the final manuscript.
